# Deep learning for detecting herbicide weed control spectrum in turfgrass

**DOI:** 10.1186/s13007-022-00929-4

**Published:** 2022-07-25

**Authors:** Xiaojun Jin, Muthukumar Bagavathiannan, Aniruddha Maity, Yong Chen, Jialin Yu

**Affiliations:** 1 College of Mechanical and Electronic Engineering, Nanjing Forestry University, Nanjing, 210037 Jiangsu China; 2 Peking University Institute of Advanced Agricultural Sciences, Weifang, 261325 Shandong China; 3 Department of Soil and Crop Sciences, Texas A&M University, College Station, TX 77843 USA

**Keywords:** Deep learning, Herbicide weed control spectrum, Precision herbicide application, Weed detection

## Abstract

**Background:**

Precision spraying of postemergence herbicides according to the herbicide weed control spectrum can substantially reduce herbicide input. The objective of this research was to evaluate the effectiveness of using deep convolutional neural networks (DCNNs) for detecting and discriminating weeds growing in turfgrass based on their susceptibility to ACCase-inhibiting and synthetic auxin herbicides.

**Results:**

GoogLeNet, MobileNet-v3, ShuffleNet-v2, and VGGNet were trained to discriminate the vegetation into three categories based on the herbicide weed control spectrum: weeds susceptible to ACCase-inhibiting herbicides, weeds susceptible to synthetic auxin herbicides, and turfgrass without weed infestation (no herbicide). ShuffleNet-v2 and VGGNet showed high overall accuracy (≥ 0.999) and F_1_ scores (≥ 0.998) in the validation and testing datasets to detect and discriminate weeds susceptible to ACCase-inhibiting and synthetic auxin herbicides. The inference time of ShuffleNet-v2 was similar to MobileNet-v3, but noticeably faster than GoogLeNet and VGGNet. ShuffleNet-v2 was the most efficient and reliable model among the neural networks evaluated.

**Conclusion:**

These results demonstrated that the DCNNs trained based on the herbicide weed control spectrum could detect and discriminate weeds based on their susceptibility to selective herbicides, allowing the precision spraying of particular herbicides to susceptible weeds and thereby saving more herbicides. The proposed method can be used in a machine vision-based autonomous spot-spraying system of smart sprayers.

## Introduction

Turf is the predominant vegetation cover in urban landscapes, such as athletic fields, institutional and residential lawns, parks, and golf courses [1]. Weeds can be a significant challenge for turf management. Weeds compete with turfgrass for environmental resources such as sunlight, water, and nutrients [2, 3], reducing turf aesthetics and functionality. Herbicides are typically broadcast-applied for weed control [4], resulting in unnecessary application of herbicide to turf areas where weeds do not occur [5, 6]. This is a source of concern because excessive use of synthetic herbicides could potentially pollute the environment [6–9]. For example, monosodium methyl arsenate (MSMA), an organic arsenical herbicide, is used to control difficult-to-control weeds in bermudagrass [*Cynodon dactylon* (L.) Pers.] turf, but is detected in underground water [10]. In the United States, only a single broadcast application of MSMA is permitted for newly constructed golf courses per year. Application of MSMA on existing golf courses is limited to spot application and should not exceed 25% of the total turf area per year [7]. However, manual spot-spraying of herbicides is time-consuming and labor-intensive, and thus is unfeasible for large turf areas.

Machine vision-based precision herbicide spraying can reduce herbicide input and weed control costs [11]. Accurate weed detection is a prerequisite for automatic precision herbicide application [12, 13]. Various visual characteristics have been studied for weed detection and classification through image processing techniques, such as color [14], morphological [15], and textural features [16]. However, none of them can reliably detect and discriminate weeds due to the fact that crops and weeds may exhibit similar morphological characteristics [2, 17]. In recent years, deep learning, especially deep convolutional neural networks (DCNNs), has made significant advancements in image classification and object detection [18, 19]. Deep learning technologies have an extraordinary ability to automatically learn representations from raw data without introducing hand-coded rules or human domain knowledge and extract complex features from images with a high accuracy level [11, 20]. It has proven to be a powerful tool in computer vision [18, 21, 22], natural language processing [23, 24], and speech recognition [25, 26].

In agriculture, previous studies demonstrated the effectiveness of using DCNNs for weed detection [27, 28], disease detection [29, 30], yield prediction [31, 32], insect damage recognition [33, 34], and crop quality examination [35–37]. A large number of studies have investigated the feasibility of using DCNNs for weed detection in various cropping systems, such as vegetable [38], corn (*Zea mays* L.) [39], soybean [*Glycine max* (L.) Merr.] [40], wheat (*Triticum aestivum* L.) [41], and turf [5, 7, 42, 43]. Kamilaris et al. concluded that deep learning techniques generally outperformed traditional image processing methods for weed detection and classification [44].

The feasibility of using deep learning technology for weed detection and classification in turf was first reported by Yu et al. [42, 43], who compared three image classification neural networks including AlexNet, GoogLeNet, and VGGNet, and found that VGGNet effectively detected various broadleaf weeds including common chickweed [*Stellaria media* (L.) Vill.], dandelion (*Taraxacum officinale* F. H. Wigg.), henbit (*Lamium amplexicaule* L.), purple deadnettle (*Lamium purpureum* L.), and white clover (*Trifolium repens* L.) growing in dormant bermudagrass [42]. In another investigation, VGGNet also effectively detected grassy weeds including crabgrass (*Digitaria* spp.), doveweed [*Murdannia nudiflora* (L.) Brenan], dallisgrass (*Paspalum dilatatum* Poir.), and tropical signalgrass [*Urochloa distachya* (L.) T.Q. Nguyen] growing in bermudagrass turf [43].

Despite all the recent successes, none of the previous studies attempted to train deep learning models for detecting and discriminating different weed species growing in turf based on their susceptibility to particular herbicides. To achieve selective herbicide spraying, the machine vision system of an automatic herbicide sprayer (carry multiple herbicides) must be able to determine the types of herbicides that need to be sprayed. Therefore, the outputs of weed species neural networks cannot be used to guide and control the sprayers directly. Effective discrimination of weed species based on the herbicide weed control spectrum allows the smart sprayer to spray particular herbicides to control the susceptible weeds, thereby saving more herbicides. Crabgrass (*Digitaria ischaemum* L.), dallisgrass, dollarweed (*Hydrocotyle* spp.), goosegrass (*Eleusine indica* L*.*), old world diamond-flower (*Hedyotis cormybosa* L*.*), tropical signalgrass, Virginia buttonweed (*Diodia virginiana* L.), and white clover are the most common turf weeds in the Southeast United States. The performances of DCNNs for detecting and discriminating these weed species in turf were evaluated with the ultimate goal of selective herbicide application based on the herbicide weed control spectrum. The objectives of this research were to (1) investigate the feasibility of using DCNNs for detecting and discriminating weeds growing in bermudagrass turf based on their susceptibility to ACCase-inhibiting and synthetic auxin herbicides, (2) evaluate and compare the performance of DCNNs for discriminating individual weed species, and (3) determine the best herbicide weed control spectrum neural network by jointly analyzing the overall accuracy, F_1_ score, and inference time.

## Materials and method

### Overview

In this study, the DCNNs were trained according to the herbicide weed control spectrum with the ultimate goal of autonomous spot-spraying herbicides. Four image classification DCNNs, including GoogLeNet [45], MobileNet [46], ShuffleNet [47], and VGGNet [48] were evaluated to detect and discriminate weeds growing in bermudagrass turf. GoogLeNet is a type of neural network in the form of inception architecture. GoogLeNet reduces the number of neurons and parameters by taking an average among the channels right before the dense layer. MobileNet is constructed based on streamlined architecture, using depth-wise separable convolutions to build lightweight neural networks. MobileNet provides efficient and low-power models for mobile devices. ShuffleNet is designed for mobile applications with minimal requirement of computing power. It utilizes pointwise group convolution and channel shuffle to reduce computation cost while maintaining accuracy. VGGNet, also known as VGG-16, is composed of 13 convolutional layers and 3 fully connected layers. It has smaller filters with more depth instead of having large filters. These DCNN architectures were used for classifying and discriminating if the sub-images contain weeds susceptible to particular herbicides or exclusively contain bermudagrass turf without weed infestation.

### Image acquisition

The training images of dallisgrass, goosegrass, Virginia buttonweed, and white clover growing in bermudagrass turf were acquired at the University of Georgia Griffin Campus in Griffin, Georgia, United States (33.26° N, 84.28° W), while the testing images were primarily taken in multiple golf courses in Peachtree City, Georgia, United States (33.39° N, 84.59° W). The training images of crabgrass, dollarweed, old world diamond-flower, and tropical signalgrass were taken at multiple golf courses in Bradenton (27.49° N, 82.47° W), Tampa (27.95° N, 82.45° W), Riverview (27.86° N, 82.32° W), and Sun City, Florida (27.71° N, 82.35° W), while the testing images were taken at multiple institutional lawns and golf courses in Lakeland, Florida (28.03° N, 81.94° W). The training and testing images of crabgrass, dallisgrass, dollarweed, goosegrass, old world diamond-flower, tropical signalgrass, Virginia buttonweed, and white clover were taken multiple times from April to November 2018 using a digital camera (DSC-HX1, SONY®, Cyber-Shot Digital Still Camera, SONY Corporation, Minato, Tokyo, Japan) at a ratio of 16:9, with an original dimension of 1920 × 1080 pixels. The camera was set on automatic modes for parameters including exposure, focus, white balance, etc. During image acquisition, the images were adjusted at a height to obtain a ground-sampling distance of 0.05 cm pixel^−1^. The images were taken from 9:00 AM to 5:00 PM under various illumination conditions, including cloudy, partly cloudy, and sunny days.

### Training and testing

**Fig. 1:**
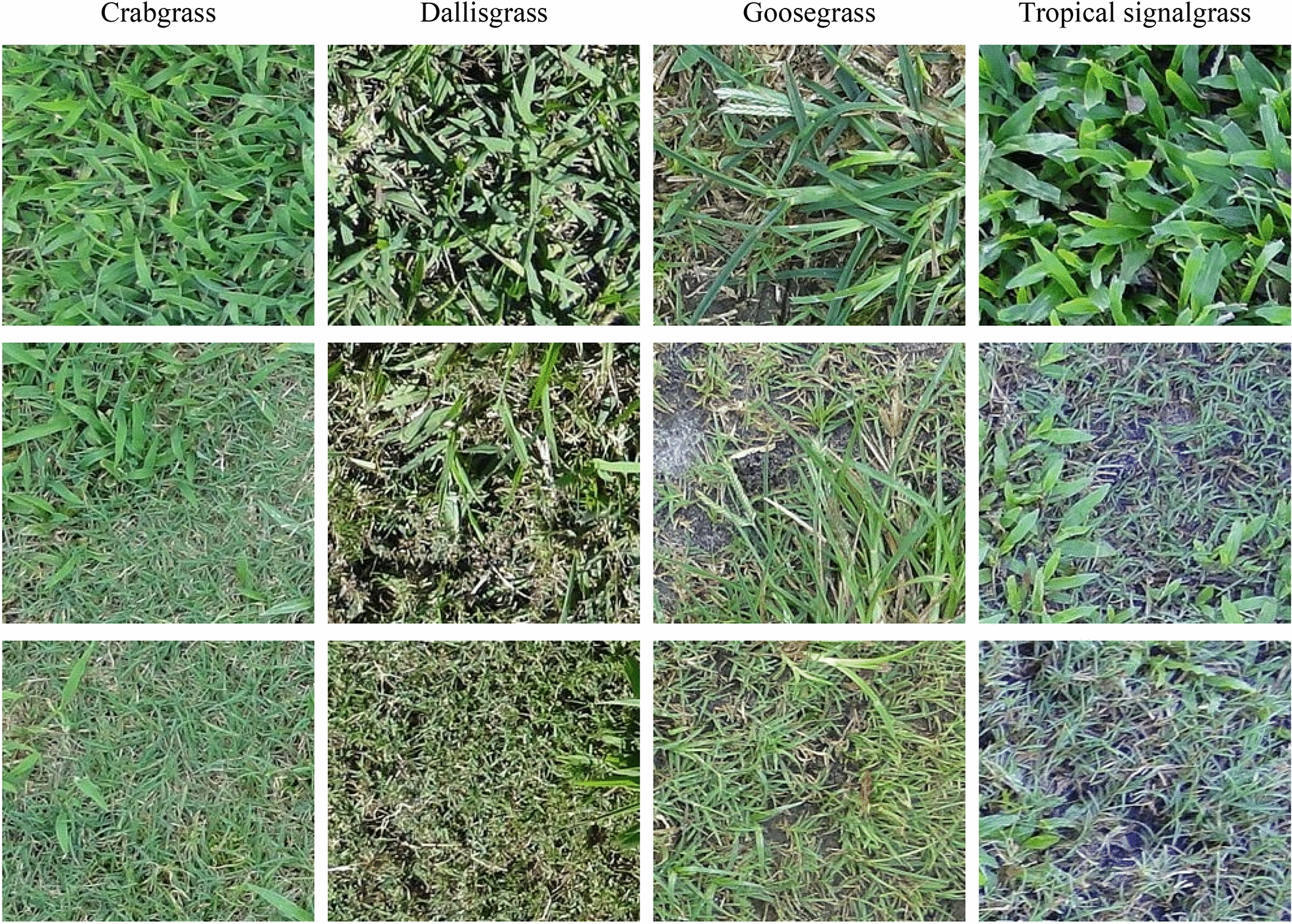
The training and testing images of crabgrass, dallisgrass, goosegrass, and tropical signalgrass at different growth stages and densities

**Fig. 2:**
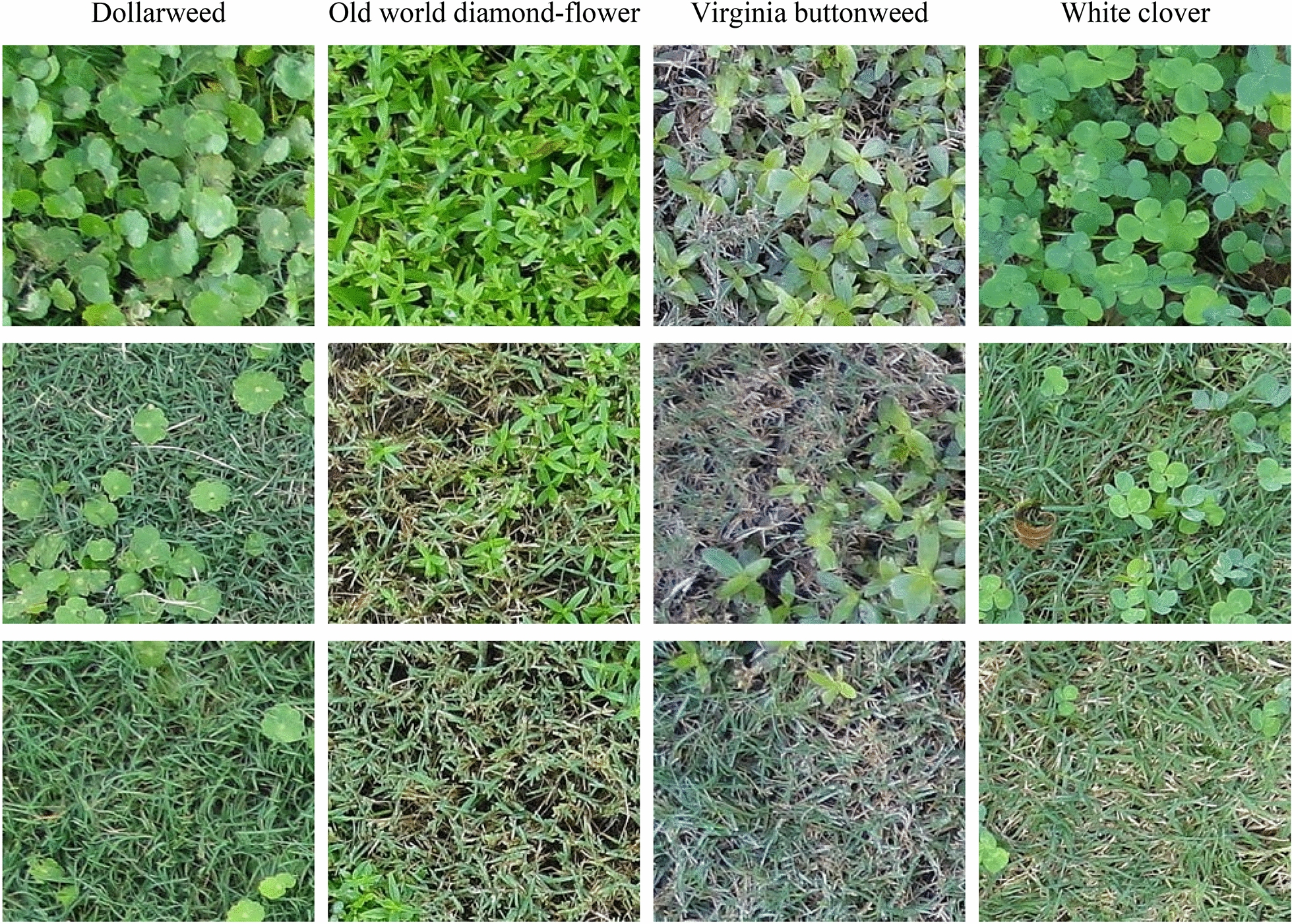
The training and testing images of dollarweed, old world diamond-flower, Virginia buttonweed, and white clover at different growth stages and densities

**Fig. 3:**
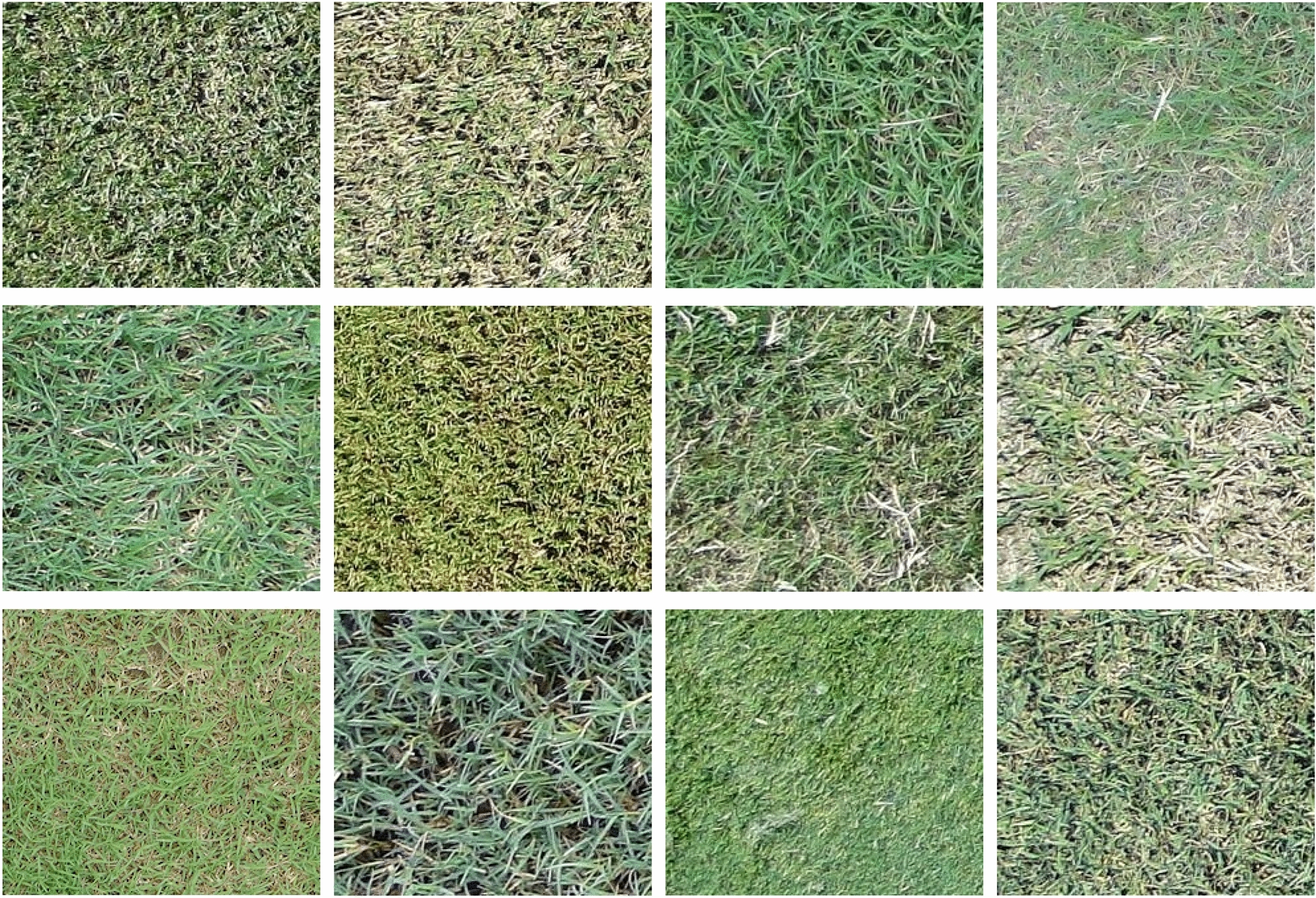
The training and testing images of bermudagrass at different turfgrass management regimes, mowing heights, and surface conditions

Images containing a single weed species were selected and used for training and testing. Images containing crabgrass, dallisgrass, dollarweed, goosegrass, old world diamond-flower, tropical signalgrass, Virginia buttonweed, and white clover growing in bermudagrass turf were cropped into 40 sub-images (5 rows × 8 columns, 40 grid cells) with a resolution of 240 × 216 pixels using ImageJ (version 2.1.0, an open-source software available at https://github.com/imagej/imagej). Sub-images of crabgrass, dallisgrass, goosegrass, and tropical signalgrass (Fig. 1), dollarweed, old world diamond-flower, Virginia buttonweed, and white clover (Fig. 2) at varying growth stages and densities, and sub-images of bermudagrass (Fig. 3) at varying turf management regimes, including different mowing heights and surface conditions were distributed evenly and used for training and testing the neural networks.

The herbicide weed control spectrum neural networks were trained using a dataset containing 3 classes of sub-images: weeds susceptible to ACCase-inhibiting herbicides, weeds susceptible to synthetic auxin herbicides, and turf without weed infestation. To constitute the training dataset of the herbicide weed control spectrum neural networks, the aforementioned sub-images containing crabgrass, dallisgrass, goosegrass, or tropical signalgrass (susceptible to ACCase-inhibiting herbicides) were randomly selected, pooled, and labeled with *ACCase-inhibiting herbicides*, the aforementioned sub-images containing dollarweed, old world diamond-flower, Virginia buttonweed, or white clover (susceptible to synthetic auxin herbicides) were randomly selected, pooled, and labeled with *Synthetic auxin herbicides*, whereas the aforementioned sub-images containing only bermudagrass turf were used as the true negative images and labeled with *No herbicide* (Table 1).

**Table 1:** The number of sub-images used to constitute the training, validation, and testing datasets of the herbicide weed control spectrum neural networks

Dataset	Weeds susceptible to ACCase-inhibiting herbicides				Weeds susceptible to synthetic auxin herbicides				No herbicide
	Crabgrass	Dallisgrass	Goosegrass	Tropical signalgrass	Dollarweed	Old world diamond-flower	Virginia buttonweed	White clover	Bermudagrass
Training	3000	3000	3000	3000	3000	3000	3000	3000	12,000
Validation	600	600	600	600	600	600	600	600	2400
Testing	600	600	600	600	600	600	600	600	2400

The herbicide weed control spectrum neural networks were trained to detect and discriminate the sub-images containing weeds susceptible to ACCase-inhibiting herbicides, weeds susceptible to synthetic auxin herbicides, or bermudagrass turf exclusively (no herbicide)

Weed species neural network was trained because we were interested in comparing the performances of the DCNNs for identifying individual weed species growing in bermudagrass turf. To constitute the training dataset of the weed species neural networks, a total of 24,000 sub-images (3000 images for each weed species) containing crabgrass, dallisgrass, dollarweed, goosegrass, old world diamond-flower, tropical signalgrass, Virginia buttonweed, or white clover growing in bermudagrass turf were randomly selected and used as the true positive images. A total of 12,000 sub-images containing bermudagrass turf exclusively were randomly selected and used as the true negative images.

To constitute the validation or testing dataset (independent of each other) of the herbicide weed control spectrum neural networks, the aforementioned sub-images containing crabgrass, dallisgrass, goosegrass, or tropical signalgrass were pooled and labeled with *ACCase-inhibiting herbicides*, the aforementioned sub-images containing dollarweed, old world diamond-flower, Virginia buttonweed, or white clover were pooled and labeled with *Synthetic auxin herbicides*, while the aforementioned sub-images containing bermudagrass turf only were used as the true negative images and labeled with *No herbicide* (Table 1). To constitute the validation or testing dataset of the weed species neural networks, a total of 4800 sub-images (600 images for each weed species) containing crabgrass, dallisgrass, dollarweed, goosegrass, old world diamond-flower, tropical signalgrass, Virginia buttonweed, or white clover growing in bermudagrass were randomly selected and used as the true positive images. A total of 2400 sub-images containing bermudagrass turf exclusively were randomly selected and used as the true negative images.

The training and testing were performed in PyTorch open-source deep learning environment (available at https://pytorch.org/; Facebook, San Jose, California, United States) using a graphic processing unit (NVIDIA GeForce RTX 2080 Ti, NVIDIA; Santa Clara, USA). The DCNNs were pre-trained using ImageNet to initialize the weights and bias through the transfer learning approach [49, 50]. The hyper-parameters used for training the DCNNs are presented in Table 2.

**Table 2:** Values of the hyperparameters for the neural networks

Deep learning architecture	Optimizer	Base learning rate	Learning rate policy	Batch size	Training epochs
GoogLeNet	Adam	0.0003	StepLR	48	60
MobileNet-v3	Adam	0.0001	StepLR	48	60
ShuffleNet-v2	SGD	0.001	LambdaLR	48	60
VGGNet	Adam	0.0001	StepLR	48	60

*SGD* stochastic gradient descent

The training and testing results of image classification DCNNs were arranged in a binary classification confusion matrix consisting of four conditions: a true positive (*tp*), a true negative (*tn*), a false positive (*fp*), and a false negative (*fn*). The performances of the DCNNs were evaluated in terms of precision, recall, overall accuracy, and F_1_ score.

Precision measures the ability of the neural network to detect the target and was calculated using the following equation [51]:

1 $$ {\text{precision}} = \frac{tp}{{tp + fp}}. $$

Recall measures the effectiveness of the neural network to detect the target and was computed using the following equation [51]:

2 $$ {\text{recall}} = \frac{tp}{{tp + fn}}. $$

Overall accuracy measures the ratio between the corrected prediction and the total observation and was defined using the following equation [51]:

3 $$ {\text{Overall}}\;{\text{accuracy}} = \frac{tp + tn}{{tp + fp + fn + tn}}.$$

The F_1_ score measures the overall performance of the neural network and was defined as the harmonic means of precision and recall, which was determined using the following equation [51]:

4 $$ {F_1} = \frac{2 \times precision \times recall}{{precision + recall}}. $$

Frames per second (FPS) measures the number of images, known as frames, are processed by the neural network per second. The higher the FPS value, the faster the image processing is. The FPS was adopted as a quantitative metric to evaluate the speed of different neural networks.

## Results and discussion

For herbicide weed control spectrum neural networks, no obvious differences were observed among GoogLeNet, ShuffleNet-v2, and VGGNet for detecting and discriminating weeds susceptible to ACCase-inhibiting and synthetic auxin herbicides (Table 3). The precision, recall, overall accuracy, and F_1_ score values of MobileNet-v3 were consistently lower than other neural networks in the validation and testing datasets. In general, the performances of herbicide weed control spectrum neural networks were slightly reduced in the testing datasets compared to the validation datasets. For detecting and discriminating the sub-images containing bermudagrass turf exclusively, the F_1_ score of MobileNet-v3 was 0.975 in the testing dataset, while the F_1_ scores of all other neural networks never fell below 0.998. ShuffleNet-v2 and VGGNet showed high overall accuracy (≥ 0.999) and F_1_ scores (≥ 0.998) in the validation and testing datasets to detect and discriminate weeds susceptible to ACCase-inhibiting and synthetic auxin herbicides.

**Table 3:** The performances of the herbicide weed control spectrum neural networks for detecting and discriminating the sub-images containing weeds susceptible to ACCase-inhibiting herbicides, weeds susceptible to synthetic auxin herbicides, or bermudagrass turf exclusively (no herbicide)

Deep learning architecture	Herbicides	Validation dataset				Testing dataset			
		Precision	Recall	Overall accuracy	F_1_ score	Precision	Recall	Overall accuracy	F_1_ score
GoogLeNet	ACCase-inhibiting	0.995	0.999	0.998	0.997	0.993	0.999	0.997	0.996
	Synthetic auxin	0.999	0.995	0.998	0.997	0.998	0.994	0.997	0.996
	No herbicide	1.000	0.999	1.000	0.999	1.000	0.999	1.000	0.999
MobileNet-v3	ACCase-inhibiting	0.976	0.965	0.980	0.970	0.973	0.963	0.979	0.968
	Synthetic auxin	0.978	0.978	0.985	0.978	0.981	0.971	0.984	0.976
	No herbicide	0.971	0.983	0.985	0.977	0.965	0.985	0.983	0.975
ShuffleNet-v2	ACCase-inhibiting	1.000	1.000	1.000	1.000	1.000	0.999	1.000	0.999
	Synthetic auxin	0.999	1.000	1.000	0.999	0.999	1.000	0.999	0.999
	No herbicide	1.000	1.000	1.000	1.000	1.000	1.000	1.000	1.000
VGGNet	ACCase-inhibiting	0.998	1.000	0.999	0.999	0.998	0.999	0.999	0.998
	Synthetic auxin	1.000	1.000	1.000	1.000	0.998	1.000	0.999	0.999
	No herbicide	1.000	0.998	0.999	0.999	1.000	0.997	0.999	0.998

The inference time is critical for real-time weed detection and precision herbicide application. The speed of weed detection, in terms of FPS, is shown in Table 4. The FPS values of the herbicide weed control spectrum neural networks were calculated using images from the testing dataset. VGGNet demonstrated a significant speed advantage (189.10fps) over the other herbicide weed control spectrum neural networks (≤ 142.15fps) when detecting and discriminating the sub-images (240 × 216 pixels) with a batch size value of 1. Since the machine vision sub-system of our developed smart sprayer prototype captures images at a resolution of 1920 × 1080 pixels, the classification speed with original images was measured (by inferring the sub-image with a batch size value of 40). When detecting and discriminating the original images, ShuffleNet-v2, with 58.21 images inferred per second, was 6.61 slower than MobileNet-v3, but noticeably faster than GoogLeNet and VGGNet. MobileNet-v3 and ShuffleNet-v2 exhibited faster inference rates and outperformed the other neural networks on classification efficiency.

**Table 4:** The inference time of the neural networks evaluated in the study

Deep learning architecture	Image type	Resolution	Batch size	FPS
GoogLeNet	Sub-image	240 × 216	1	140.97
	Image	1920 × 1080	40	34.46
MobileNet-v3	Sub-image	240 × 216	1	142.15
	Image	1920 × 1080	40	64.82
ShuffleNet-v2	Sub-image	240 × 216	1	133.22
	Image	1920 × 1080	40	58.21
VGGNet	Sub-image	240 × 216	1	189.10
	Image	1920 × 1080	40	8.76

*FPS* frames per second

**Fig. 4:**
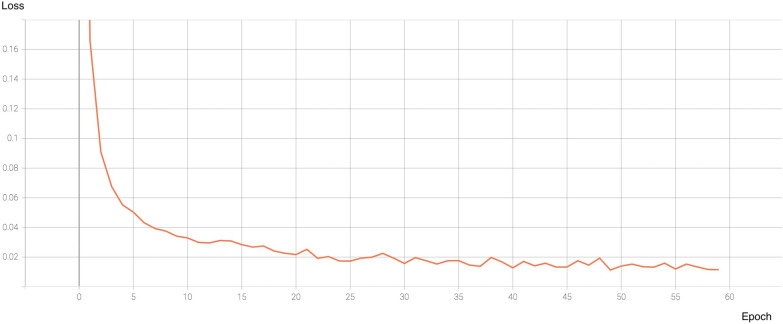
The learning curve of ShuffleNet-v2 when it was trained to detect herbicide weed control spectrum

By jointly analyzing the overall accuracy, F_1_ score, and FPS, ShuffleNet-v2 demonstrated superiorities in both accuracy and computational efficiency compared to the other herbicide weed control spectrum neural networks. This competitive result may mainly come from implementing pointwise group convolution and channel shuffle [47]. Overall, these results demonstrated that ShuffleNet-v2 was the most efficient and accurate model for detecting and discriminating weeds growing in turf susceptible to ACCase-inhibiting and synthetic auxin herbicides. Figure 4 shows the learning curve of ShuffleNet-v2 over 60 training epochs. The value of the loss function changes with training epochs, which forms the loss curve. The loss value quickly approaches 0.05 after 5 epochs. The loss curve continues to decline and stabilize, indicating minimal overfitting.

Table 5 presents the metrics results when ShuffleNet-v2 was trained to detect and discriminate individual weed species. ShuffleNet-v2 exhibited excellent overall accuracy (≥ 0.997) and F_1_ score (≥ 0.980) with high precision and recall values in the validation datasets for detecting and discriminating the sub-images containing dallisgrass, goosegrass, old world diamond-flower, or Virginia buttonweed growing in bermudagrass turf and the sub-images containing bermudagrass turf exclusively. ShuffleNet-v2 had slightly reduced precision, recall, overall accuracy, and F_1_ score values in the testing dataset. For detecting and discriminating crabgrass, dollarweed, tropical signalgrass, or white clover, the F_1_ score of ShuffleNet-v2 never exceeded 0.932 in the validation and testing datasets, although it is the best herbicide weed control spectrum neural network.

**Table 5:** Weed detection validation and testing results when ShuffleNet-v2 was trained to detect and discriminate individual weed species

Deep learning architecture	Weed species	Validation dataset				Testing dataset			
		Precision	Recall	Overall accuracy	F_1_ score	Precision	Recall	Overall accuracy	F_1_ score
ShuffleNet-v2	Bermudagrass	1.000	1.000	1.000	1.000	1.000	1.000	1.000	1.000
	Crabgrass	0.923	0.942	0.989	0.932	0.915	0.937	0.988	0.926
	Dallisgrass	0.990	0.970	0.997	0.980	0.985	0.970	0.996	0.977
	Dollarweed	0.923	0.913	0.986	0.918	0.922	0.903	0.986	0.912
	Goosegrass	0.971	0.990	0.997	0.980	0.969	0.985	0.996	0.977
	Old world diamond-flower	0.984	0.997	0.998	0.990	0.980	0.998	0.998	0.989
	Tropical signalgrass	0.940	0.918	0.988	0.929	0.935	0.910	0.987	0.922
	Virginia buttonweed	0.995	0.983	0.998	0.989	0.995	0.980	0.998	0.987
	White clover	0.913	0.923	0.986	0.918	0.903	0.920	0.985	0.911

**Fig. 5:**
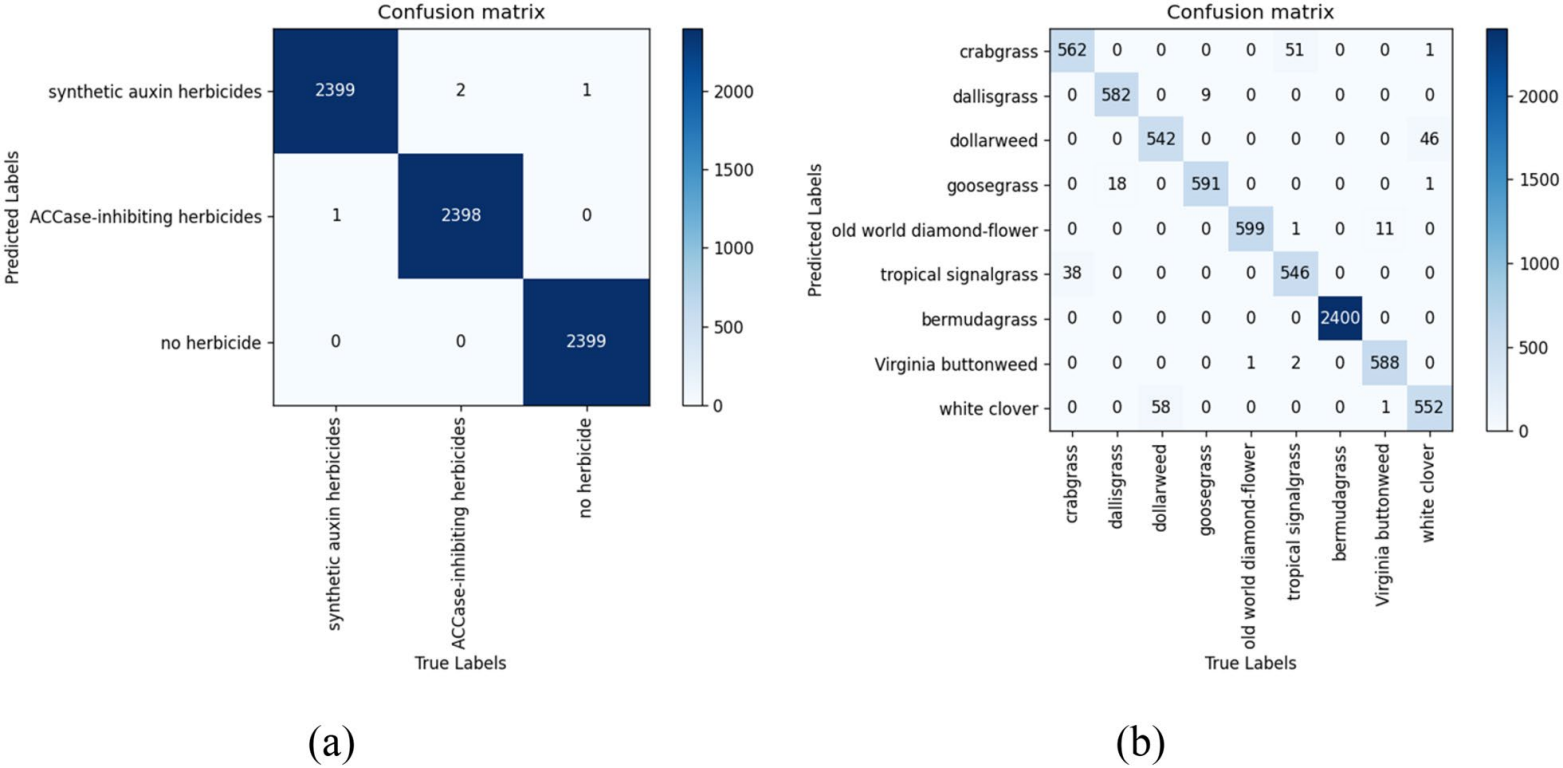
Confusion matrices when ShuffleNet-v2 was trained as herbicide weed control spectrum neural network (**a**) and weed species neural network (**b**), respectively

ShuffleNet-v2 presented a superiority in detecting the susceptibility of weed species to herbicides (Fig. 5). It was observed that 51 tropical signalgrass were misclassified as crabgrass, 18 dallisgrass were misclassified as goosegrass, 58 dollarweed were misclassified as white clover, and 11 Virginia buttonweed were misclassified as old world diamond-flower in the testing dataset. These weed species are morphologically similar. Therefore, it can be deduced that training DCNN models according to the herbicide weed control spectrum would likely eliminate the similarity issue in weed morphology and thereby increase detection accuracy.

In the present study, weed vegetation was only discriminated into two categories: weeds susceptible to ACCase-inhibiting herbicides versus weeds susceptible to synthetic auxin herbicides. While the herbicide weed control spectrum neural networks achieved high classification rates, more positive images of the training dataset comprised of three or even more categories of herbicides are highly desired. An additional study is needed to evaluate the feasibility of detecting and discriminating three weed vegetation categories, including broadleaf, grass, and nutsedge weeds growing in turf.

It should be noted that diclofop-methyl is the only ACCase-inhibitor that can be used to selectively control grass weeds, such as goosegrass and ryegrass (*Lolium* spp.), in bermudagrass turf [4, 52], while other ACCase-inhibitors such as fenoxaprop and fluazifop (aryloxyphenoxypropionate) are used to control grassy weeds in cool-season turfgrasses, and zoysiagrass (*Zoysia* spp.) [53, 54], and sethoxydim (cyclohexanedione) is used to control grassy weeds in centipedegrass [*Eremochloa ophiuroides* (Munro) Hack.] [55]. The majority of synthetic auxin herbicides (e.g. 2,4-D, dicamba, and mecoprop) are postemergence herbicides that selectively control broadleaf weeds within bermudagrass turf with only a few exceptions [4, 56, 57]. For example, quinclorac controls both broadleaf and crabgrass weeds in bermudagrass turf, while triclopyr is used to suppress bermudagrass in cool-season turfgrasses [58–60].

In this study, all training and testing images were cropped into 40 sub-images (grid cells). The image classification DCNNs were trained using these sub-images with a resolution of 240 × 216 pixels. Each sub-image (grid cell) represented a physical size of 10 cm × 9 cm. In a practical machine vision system, a custom software will be utilized to build a grid cell map and detect the location of weeds on the input image by identifying if the grid cells contain weeds that are susceptible to particular herbicides. The resolution of the sub-image (physical size) should be equal to or slightly smaller than the size of the area in which one nozzle is covered. In the future study, the trained herbicide weed control spectrum neural networks are employed to infer if the grid cells contained weeds. The grid cells are marked as spraying areas if the inference indicates they contain weeds. With a subsequent decision-making system, only the nozzles corresponding to those cells infested with weeds susceptible to selective herbicides are turned on, thus realizing smart sensing and spraying.

It should be noted that weeds susceptible to ACCase-inhibiting herbicides may be misclassified as susceptible to synthetic auxin herbicides (or vice versa) during field applications; however, this is unlikely to be an issue because areas with weed infestation have been detected. The occurrence of this type of erroneous classification can be minimized by increasing the number of training images containing such weed species.

Discriminating different categories of weed species growing in turf based on their susceptibility to selective herbicides allows spraying particular herbicides for weed control, thereby saving more herbicides. It should be noted that the weed species examined in the present study are the most common turf weeds in the Southeast United States. The purpose of the training dataset is to learn representations of different weeds and complex field environments on the performance of deep learning models applied to natural images. Improving the robustness and adaptability of the developed herbicide weed control spectrum neural networks depends on obtaining diverse training data. An additional study is needed to include a more diverse weed species in the training and testing datasets. Based on the high-level performance, the proposed method is highly suitable for ground-based weed detection in turf.

## Summary and conclusions

This work demonstrated the feasibility of using image classification DCNNs to detect and discriminate weeds growing in bermudagrass turf based on their susceptibility to ACCase-inhibiting and synthetic auxin herbicides. This is the first study attempting to train DCNNs for detecting and discriminating weeds based on their susceptibility to selective herbicides, which will allow the use of particular herbicides for precision spraying susceptible weeds to save more herbicides.

ShuffleNet-v2 and VGGNet showed high overall accuracy (≥ 0.999) and F_1_ scores (≥ 0.998) in the validation and testing datasets to detect and discriminate weeds susceptible to ACCase-inhibiting and synthetic auxin herbicides. ShuffleNet-v2 was the best herbicide weed control spectrum neural network as it exhibited higher accuracy and computational efficiency among the neural networks evaluated. ShuffleNet-v2 presented a superiority in discriminating weeds based on their susceptibility to herbicides compared to when it was used to detect and discriminate individual weed species. The developed herbicide weed control spectrum neural network can be used in a machine vision sub-system with an automatic herbicide sprayer to achieve selective herbicide spraying.

## Data Availability

The datasets used in this study is available from the corresponding author on reasonable request.

## Abbreviations

DCNNs: Deep convolutional neural networks
MSMA: Monosodium methyl arsenate
FPS: Frames per second
